# A Case of Secondary Extramammary Paget's Disease Surrounding a Cutaneous Ureterostoma After Recurrence of Bladder Urothelial Carcinoma

**DOI:** 10.1002/iju5.70021

**Published:** 2025-03-27

**Authors:** Masahiro Mizoguchi, Ichiro Chihara, Keisuke Sano, Tomokazu Kimura, Shuya Kandori, Hiromitsu Negoro, Bryan J. Mathis, Hitomi Kawai, Daisuke Matsubara, Hiroyuki Nishiyama

**Affiliations:** ^1^ Department of Urology Institute of Medicine, University of Tsukuba Tsukuba Ibaraki Japan; ^2^ Department of Cardiovascular Surgery Institute of Medicine, University of Tsukuba Tsukuba Ibaraki Japan; ^3^ Department of Pathology Institute of Medicine, University of Tsukuba Tsukuba Ibaraki Japan

**Keywords:** bulging, erythema, Paget disease extramammary, ureterocutaneostomy, urothelial carcinoma

## Abstract

**Introduction:**

Secondary extramammary Paget's disease features intraepidermal carcinoma caused by tumor invasion and migration into adjacent skin.

**Case Presentation:**

A 68‐year‐old man underwent radical cystectomy and bilateral cutaneous ureterostomy for bladder cancer and right ureteral cancer. Postoperative follow‐up computed tomography and urine cytology at 3 years suggested right ureteral cancer recurrence. Contemporaneous erythema and bulging of the skin around the right ureterocutaneous fistula were observed, necessitating retroperitoneoscopic nephroureterectomy and peristomal skin resection. Pathology revealed urothelial carcinoma with T2 disease at the lower ureter, plus spreading cytokeratin 7+ and cytokeratin 20+ pagetoid cells clustered within the epidermis indicative of secondary Paget's disease.

**Conclusion:**

In cases of recurrent upper urothelial carcinoma after urinary diversion, patients may develop secondary extramammary Paget's disease of the skin.

**Summary**

- Secondary extramammary Paget's disease (SEMPD) around a cutaneous ureterostoma is rare.
- Regular monitoring of skin lesions around the stoma offers an early, noninvasive detection method.
- SEMPD should be considered in the differential diagnosis if tumor formation or refractory dermatitis is present.

**Abbreviations**
CK20: cytokeratin 20
CK7: cytokeratin 7
CT: computed tomography
EMPD: extramammary Paget's disease
GCDFP15: Gross Cystic Disease Fluid Protein‐15
HE: hematoxylin–eosin
PD: Paget's disease
SEMPD: secondary extramammary Paget's disease
UC: urothelial carcinoma

## 1 Introduction

Secondary extramammary Paget's disease (SEMPD) stems from skin‐adjacent organ cancer that migrates through the epithelium to the epidermis, presenting as intraepidermal carcinoma [1]. SEMPD skin manifestations are characterized by velvety, erythematous eczema (often mistaken for eczema or contact dermatitis) [2], usually warranting surgical resection, although standards of care remain unestablished due to disease rarity [1, 3].

Cases of urothelial carcinoma (UC)‐mediated SEMPD at the stoma site are very rare. Here, we report such a case in the right stoma site at the right ureter recurrence after radical cystectomy via bilateral cutaneous ureterostomy.

## 2 Case Presentation

A 68‐year‐old man with chronic kidney disease (estimated glomerular filtration rate 57.3 mL/min/1.73 m^2^) was diagnosed with bladder cancer (cT2N0M0) and right lower ureteral cancer (cT2N0M0) based on transurethral resection of the bladder tumor and CT. Ureteroscopy showed no tumors beyond the right lower ureter. As lower ureterectomy for lower ureteral cancer is reportedly similar in results to radical nephroureterectomy [4], he hoped to reduce dialysis risk by undergoing cystectomy, right lower ureterectomy, and bilateral ureterostomies. The intraoperative frozen section of the ureter was margin‐negative. The bladder and ureter tumors were histologically diagnosed as UC, pT3a + is N0 and UC, pT1 (3 cm from the right ureteral transection, RM0), respectively. Postoperative adjuvant chemotherapy was skipped since neoadjuvant chemotherapy (cisplatin and gemcitabine) was previously administered and other treatments were unavailable in Japan at that time.

**FIGURE 1:**
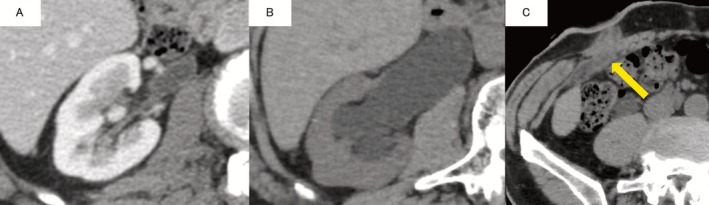
Postoperative CT findings. The patient had mild hydronephrosis after surgery (A) but this had progressed 3 years later (B). In addition, subcutaneous wall thickening and periureteral involvement were observed near the opening of the right ureter (C).

**FIGURE 2:**
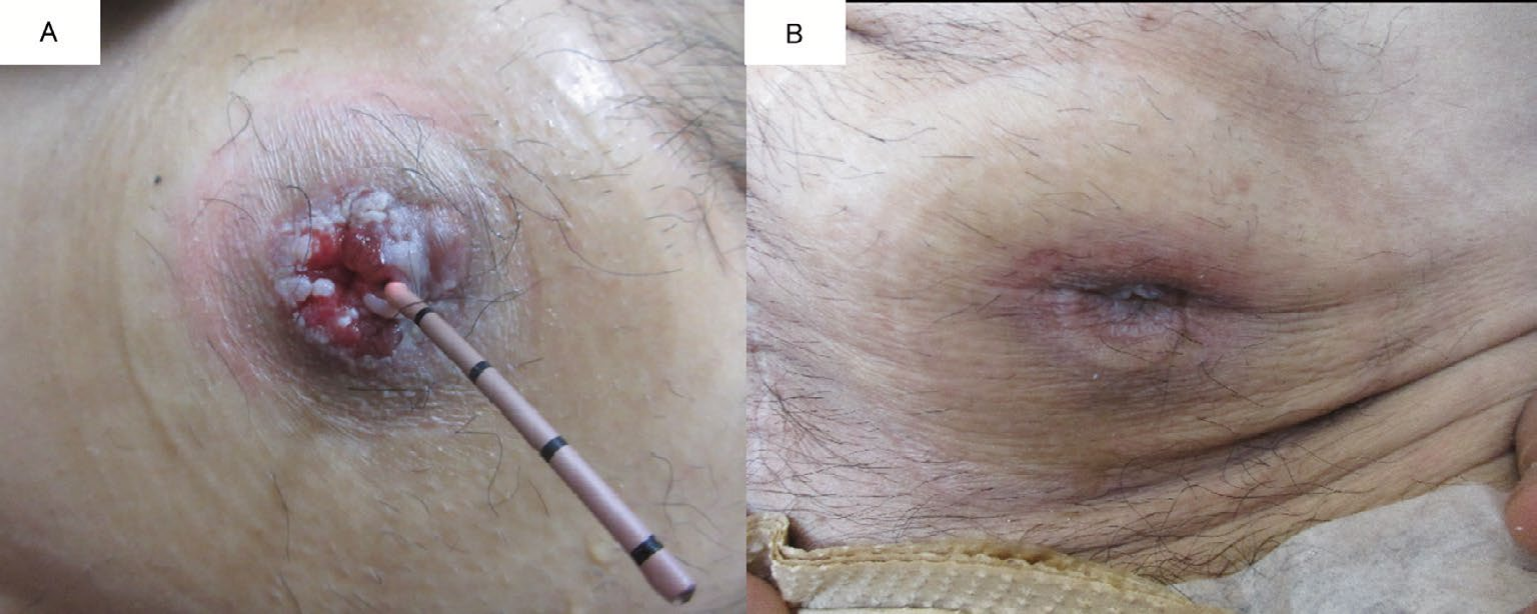
Cutaneous ureterostomy findings, revealing mild erythema and bulging around the right side (A). There were no remarkable findings on the left side (B).

**FIGURE 3:**
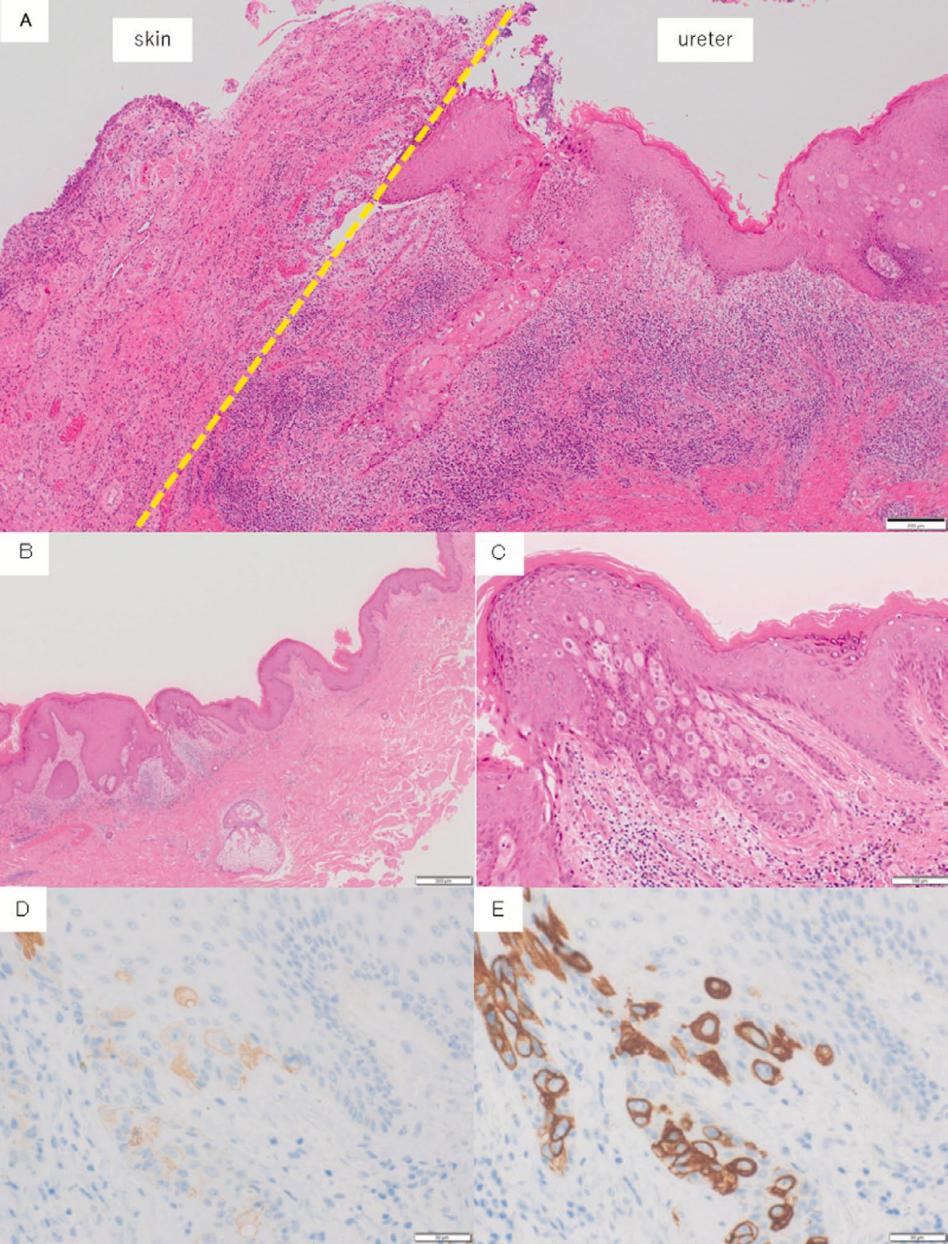
(A) The pathology of the transition area between the skin (left) and ureter (right). On the skin side, Paget cells were observed, while on the ureteral side, findings of urothelial carcinoma were present. However, no continuity is visible between these two areas. (B–E) Skin lesion pathological findings. (B) Hematoxylin–eosin findings (HE staining, magnification: 4×). (C) Large atypical cells with pale spores in the epidermis, putatively identified as Paget cells (HE staining, magnification: 20×). Paget cells in the stoma site were verified positive for both CK7 (D) and CK20 (E).

Postoperative 3‐year follow‐up CT showed right hydronephrosis and wall thickening of the right lower ureter with subcutaneous periureteral involvement (Figure 1). Simultaneous mild erythema and bulging around the right stoma were noted (Figure 2) and renal pelvic lavage cytology was class III. Right ureteral carcinoma recurrence was diagnosed based on these findings and a retroperitoneoscopic right nephroureterectomy with peristomal skin resection was performed. The ureteral tumor was located just below the right cutaneous ureterostomy, but without skin continuity, and pathology indicated UC, pT2 (Figure 3A). Although urothelial carcinoma was absent in the concurrently resected skin, hematoxylin–eosin (HE) staining (Figure 3B,C) revealed CK7+ (Figure 3D) and CK20+ (Figure 3E) tumor cells showing pagetoid spreading within the epidermal skin lesions. Finally, a diagnosis of SEMPD was made based on continuous invasion of the ureteral tumor through to the skin along with the pagetoid spreading.

He was discharged without complications and remains recurrence‐free 3 years after right nephroureterectomy.

## 3 Discussion

Paget's disease (PD) is characterized by proliferation and development of atypical large cells (Paget cells) in the epidermis and classified into breast or extramammary Paget's disease (EMPD) based on the origin site [5]. EMPD usually erupts at apocrine gland‐enriched sites [3, 6, 7, 8, 9, 10], such as the vulva, axilla, and perianal area [11]. Conversely, as SEMPD manifests from skin‐adjacent organ malignancies that tunnel through the epithelium to the epidermis, it differs from EMPD in primary lesion classification (i.e., cutaneous vs. other malignancies). Although UC may cause Paget's phenomenon at the external urethral meatus [1, 8], similarly to uterine carcinoma at the vaginal vestibule [12] or recto‐anal carcinoma at the anus [13], it only occurs in 2% of vulvar cancers and less than 1% of anal cancers [1, 13]. The frequency remains unrevealed in UC patients.

Since Paget cells may resemble pagetoid spreading tumor cells, HE staining alone is inadequate for accurate diagnosis. Typically, primary PD is CK7+ and CK20−, while SEMPD of UC origin is CK7+ and CK20+ [9]. Nevertheless, since immunostaining results may differ by cancer type, Gross Cystic Disease Fluid Protein‐15 (GCDFP15; an apocrine epithelium‐specific tissue protein) is a preferred marker for SEMPD [13] and can readily differentiate primary and secondary cancers in initially detected skin lesions [14].

Table 1 summarizes the existing SEMPD case literature involving patients with UC [15], all diagnosed as SEMPD by immunostaining for CK7/20 or GCDFP15. One case had an ileal conduit and five had cutaneous ureterostomies. Recurrence ranged from 2 to 13 years (median 4.5 years), with skin biopsies performed in all cases except ours, followed by surgery. Of note, there were obvious skin findings (i.e., tumor formation and refractory dermatitis) in all cases (including ours) (Table 1). Both early and noninvasive, regular skin observation around the stoma after urinary tract changes is an excellent prophylactic measure. However, SEMPD is rare enough to render a definitive diagnosis difficult based on skin findings alone because of differential diagnoses. Here, biopsy was eschewed because of the lower tumor localization requiring a total resection of the right renal ureter along with the concomitant resection of the skin lesion. However, all cases, except ours, were biopsied for diagnosis due to skin‐isolated findings. Thus, indeterminate skin findings should precipitate immediate biopsy instead of waiting for follow‐up.

**TABLE 1:** Extant cases with secondary extramammary Paget's disease around the urinary stoma [10, 15, 16, 17].

Report	Age, Sex	Primary tumor	Urinary diversion	Recurrence period	Presence of metastases at diagnosis	Gross findings of the stoma	Treatment of pagetoid spread	Outcome	Biopsy	Stage	Subtype	CK7	CK20	GCDFP‐15
Nakata (2010)	78, M	Ureteral cancer	Ileal conduit	13 years	NA	Dermatitis	Skin excision	NA	+	NA	NA	+	+	NA
Ito (2013)	69, F	Left ureteral cancer Tis, bladder cancer Tis	Ureterocutaneostomy	2 years	None	Tumor formation	Skin excision +right lower ureterectomy	NA	+	pTis	−	+	+	NA
Ishida (2013)	77, M	Bladder cancer T4	Ureterocutaneostomy	4 years 6 months	None	Tumor formation	Skin excision +right nephroureterectomy	NA	+	pTis	−	+	+	−
Sakatani (2013)	84, M	Prostatic urethra Tis	Ureterocutaneostomy	4 years 6 months	NA	Dermatitis	Skin excision +right nephroureterectomy	Survived for 1 year	+	pT1	−	+	+	−
Kanda (2016)	85, M	Right ureteral cancer T2, bladder cancer T2	Ureterocutaneostomy	4 years 6 months	None	Erosion	Skin excision +left lower ureterectomy	Died after 7 months	+	pT1	−	+	+	NA
Present case	68, M	Right ureteral cancer T2, bladder cancer T3a + is	Ureterocutaneostomy	3 years	None	Dermatitis	Skin excision +right nephroureterectomy	Survived for 1 year and 6 months (cancer death)	−	pT2	−	+	+	NA

While clinical guidelines to treat SEMPD remain unpublished, several reports recommend surgical resection [3, 6] of the skin lesion plus treatment of the primary lesion. Since lesions rarely extend into the dermis, segmental resection of the fatty layer should maintain 1–2 cm margins [3, 7, 18, 19]. If a 2 cm resection margin appears impossible, skin biopsy should be considered to confirm resection margins [19, 20]. Alternatively, chemotherapy targeting the primary tumor has been reported as effective for SEMPD lesions since platinum‐based chemotherapy achieved temporary lesion control in a UC case [1].

In conclusion, since SEMPD in UC patients is rare, clinicians may never encounter it after urinary tract diversion. But, until chemotherapy efficacy for SEMPD is verified, timely detection and complete resection are considered the most effective treatment. Regular observation of any skin lesions around the stoma are an excellent method of detecting SEMPD, with surgery considered if confirmed, while biopsies should be immediately conducted if skin findings are unclear.

## Consent

Patient consent for publication was obtained.

## Conflicts of Interest

The authors declare no conflicts of interest. Hiromitsu Negoro and Hiroyuki Nishiyama, Editorial Board members of *International Journal of Urology Case Reports* and co‐authors of this article, were excluded from editorial decisions on its acceptance to avoid bias.
